# Adipose stem cell-derived exosomes circular RNA circFryl attenuate atrial fibrosis and cardiomyocyte apoptosis in atrial fibrillation 

**DOI:** 10.22038/ijbms.2025.84766.18341

**Published:** 2025

**Authors:** Chunpu Li, Jiuting Tan, Xintao Deng

**Affiliations:** 1 Department of Cardiology, Xinghua People’s Hospital Affiliated to Yangzhou University, Xinghua, Jiangsu 225700, China

**Keywords:** Atrial fibrillation, Exosomes, Mesenchymal stem cells, MicroRNAs, Tissue Inhibitor of metalloproteinase-4

## Abstract

**Objective(s):**

Atrial fibrillation (AF) is a prevalent arrhythmia accompanied by structural and electrical remodeling of the heart. Here, we examined the possible mechanisms behind the protective role of adipose-derived stem cell (ADSC)-derived exosomes in AF therapy.

**Materials and Methods:**

We isolated exosomes from ADSCs. Exosome treatment was given. The left atrial diameter was measured by echocardiographic imaging. Cardiac fibrosis and damage were detected. The interaction between miR-338-3p with circFryl and tissue inhibitor of metalloproteinase mRNA (4TIMP4 mRNA) was predicted and investigated using qPCR and western blotting assay.

**Results:**

The overexpression of circFryl in ADSCs elevated the level of circFryl in exosomes and the myocytes, whereas knockdown of circFryl exhibited the opposite effects. Treatment with ADSC-exosomes significantly elevated circFryl level and recovered left atrial diameter, whereas knockdown of circFryl in exosomes abolished these effects. ADSC-exosomes alleviated the cardiac fibrosis and cell apoptosis in the AF model, and the knockdown of circFryl abolished these effects. ADSC-exosomes treatment suppressed viability and fibrosis and enhanced cell apoptosis in Ang-II-induced fibroblasts, which was reversed by depletion of circFryl. Online analysis of miRNA interaction targets showed potential binding between miR-338-3p with circFryl and TIMP4 mRNA. Knockdown of circFryl notably suppressed TIMP4 level, and inhibition of miR-338-3p recovered TIMP4 level. TIMP4 overexpression and miR-338-3p inhibition abolished the effects of sicircFryl *in vitro* and *in vivo*.

**Conclusion:**

The ADSC-derived exosomes delivered circFryl to interact with miR-338-3p, subsequently enhancing TIMP4 mRNA stability and expression in cardiac fibroblasts and myocytes. The circFryl/miR-338-3p/TIMP4 axis mediated the protective effects of ADSC on AF.

## Introduction

Atrial fibrillation (AF) is a highly prevalent cardiac arrhythmia with significant implications from both health and socio-economic perspectives (1). Apart from deteriorating patient survival and mortality, AF is associated with increased risks of stroke, dementia, and *de novo* heart failure (2, 3). Despite extensive efforts to elucidate the molecular and cellular mechanisms of AF, its complexity as an arrhythmia has impeded progress in research (4). Newly designed drugs specifically for AF treatment still face challenges, and patients, especially those with persistent AF, often rely on older antiarrhythmic drugs such as amiodarone, sotalol, propafenone, and flecainide, which exhibit limited efficacy and significant side effects (5, 2). Enhancing our understanding of the mechanisms underlying AF and identifying modulatory pathways are crucial for preventing its persistent presence. Numerous studies suggest a substantial genetic component in AF cases within the general population, surpassing traditional risk factors (6, 7).

Adipose-derived stem cells (ADSCs) hold great promise for use in trauma repair (8, 9). Extracellular vesicles, especially the exosomes, with diameters ranging from 30 to 150 nm, mediate long-distance communication between cells (10, 11). Exosomes can deliver signaling molecules such as proteins, RNA, and DNA to recipient cells (12). Compared to stem cell therapy, exosomes can reduce immune-mediated rejection reactions and malignant transformation (13, 14). Interestingly, ADSCs-derived exosomes participate in various biological processes by influencing tissue responses to injury, infection, and disease (12). ADSCs secrete more exosomes in low-oxygen environments, and hypoxia-preconditioned ADSCs-exosomes can improve blood perfusion and transplanted tissue viability and reduce inflammatory infiltration in adipose tissue (15, 16). On the other hand, various microRNAs (miRNAs) within exosomes, in particular, demonstrate the ability to promote wound healing in diabetic injuries (17, 18). 

In recent studies, exosomes produced from stem cells have been shown to have therapeutic promise for the regeneration and repair of heart tissue. ADSCs and other mesenchymal stem cells (MSCs) release exosomes that contain bioactive substances such as proteins, lipids, and microRNAs that can affect cellular responses and mediate intercellular communication. It has been documented that these exosomes promote angiogenesis, reduce oxidative stress, control immunological responses, and aid in the preservation and repair of the heart. The underlying mechanisms for their therapeutic effects include PI3K/Akt signaling, which promotes cardiomyocyte survival; TGF-β and Smad signaling, which inhibit fibrosis; and NF-κB activity suppression, which reduces inflammation. Further, exosomal cargo like miR-21 and miR-146a has been linked to apoptosis reduction and endothelial function improvement in ischemic heart disease models. Based on these favorable observations, ADSC-derived exosomes provide a new, cell-free therapeutic solution for cardiovascular disorders, including AF, by interfering with pathological remodeling processes at the molecular level (19).

In this study, we aimed to investigate the protective benefits of exosomes produced from ADSCs in AF and pinpoint the molecular processes that underlie these effects. While previous research has demonstrated the ability of stem cell-derived exosomes to regenerate in cardiovascular illness, nothing is known about the function of ADSC-exosomal circular RNAs (circRNAs) in AF. We hypothesized that exosomal circFryl generated from ADSCs might alter regulatory networks to counteract AF-associated disease, given the critical role non-coding RNAs—especially circRNAs—play in cardiac remodeling and fibrosis. In order to verify this hypothesis, we examined the effects of ADSC-derived exosomes on atrial fibrosis, cardiomyocyte apoptosis, and left atrial function using both *in vitro* and *in vivo* AF models. We also investigated circFryl, miR-338-3p, and TIMP4 interactions to better perceive their respective functions in regulating fibroblast and cardiomyocyte activity. The practical aim of this research is to bridge the knowledge gap toward understanding the potential role of ADSC-exosomal circRNAs in AF etiology and their potential as an innovative therapeutic avenue for AF by combining molecular, histological, and functional exploration.

## Materials and Methods

### Materials and reagents

This study used DMEM/F12 complete media, fetal bovine serum (FBS), and antibiotics for cell culture. Exosomes were isolated using an ultracentrifuge (Beckman, Germany) and labeled with PKH26 dye (Sigma, USA), with characterization via TEM, NTA, and confocal microscopy (Olympus, Japan). Animal models involved C57BL/6J mice (Huafukang, China) treated with acetylcholine (Macklin, China) and calcium chloride (Macklin, China), and cardiac function was assessed using ultrasound imaging (FUJIFILM VisualSonics, Canada). Histological and apoptosis assays included H&E, Masson’s trichrome, TUNEL (Beyotime, China), and microscopy (Olympus, Japan). Molecular assays used TRIzol (Invitrogen, USA), qPCR reagents (Takara, Japan), RIPA buffer, BCA kit (Beyotime, China), SDS-PAGE, western blotting, and ECL reagent (Millipore, Germany). Cell assays included CCK-8 (SolarBio, China), Annexin V/PI (Beyotime, China), and flow cytometry (Beckman, USA). Gene silencing was performed using siRNA for circFryl and miR-338-3p inhibitors.

### Identification of adipose mesenchymal stem cells (ADSCs)

ADSCs were isolated from adipose tissues of healthy adult C57BL/6J mice and cultured in DMEM/F12 complete media supplemented with 10% FBS in a humidified atmosphere with 5% CO_2_. The surface biomarkers of ADSCs, including CD44, CD90, and CD105 (positive markers) and CD106, CD34, and CD45 (negative markers), were identified using flow cytometry and immunofluorescence staining. 

### Exosome isolation and labeling

Exosomes were obtained from the supernatant of ADSCs via ultracentrifugation. In short, the cell culture supernatant underwent sequential centrifugation steps at 300×g for 10 min, 2,000×g for 10 min, and 10,000×g for 30 min, followed by filtration through a 0.22 μm filter to remove cells, dead cells, and cellular debris. For exosome purification, the supernatant was subjected to ultracentrifugation at 100,000×g for 70 min, followed by washing with PBS and another centrifugation at 100,000×g for 70 min using an Ultracentrifuge (Beckman, Germany). The size of exosomes was measured by transmission electron microscope (TEM) and nanoparticle tracking analysis (NTA). The exosomes were labeled with PKH26 (Sigma, USA) and incubated with cardiomyocytes for 24 hr. Then, images were taken by a confocal fluorescence microscope (Olympus, Japan) (20).

### Atrial fibrillation (AF) model

Eight-week-old male C57BL/6 J mice were purchased from Huafukang (Beijing, China). Five experimental groups were used: 1) Control group: healthy mice that did not receive any treatment; 2) AF model group: mice that were injected with acetylcholine (Ach) and CaCl₂ to induce AF; 3) AF + ADSC-exosome treatment group: AF mice that received injections of exosomes derived from ADSCs that depleted circFryl; 4) AF + circFryl-knockdown exosome group: AF mice that received exosomes derived from ADSCs that depleted circFryl; and 5) AF + TIMP4 overexpression/miR-338-3p inhibition group: mice that were treated with TIMP4 overexpression or miR-338-3p inhibitors in addition to circFryl-knockdown exosomes. Each group consisted of at least six mice, and treatments were administered daily for seven days. The AF model was established via intravenous injection of 60 μg/ml Ach (Macklin, Shanghai, China) and 10 mg/ml CaCl_2_ (Macklin, Shanghai, China) every day for 7 days. For treatment, the mice were injected with exosomes (50 µg/20 g body weight) and/or oligonucleotides (20 nmol/20 g body weight) every day for seven days. Mice were treated with targeted oligonucleotides to explore the molecular mechanisms. The oligonucleotides used in this work were TIMP4 overexpression plasmids (5’-UUUGUUAUUUUUGAAAUGCUGGA-3’), miR-338-3p (3’-GUUGUUUUAGUGACUACGACCU-5’) mimics and inhibitors, and small interfering RNA (siRNA) for circFryl (si-circFryl) 5’-UCUUCUCGACACUAUAUGCUGGG-3’. Each oligonucleotide was injected intravenously once daily for seven consecutive days at 20 nmol/20 g body weight. These oligonucleotides were chosen to examine the circFryl/miR-338-3p/TIMP4 axis in modulating AF-related cardiac remodeling. After model establishment, echocardiography was performed. The animal experiments were approved by the Ethics Committee of Xinghua People’s Hospital and conducted following the Guideline for Animal Care and Use (21).

### Echocardiographic measurements

Cardiac structure and function were measured by transthoracic echocardiography at baseline (Day 0) and Day 7 after establishment of the AF model. Mice were anesthetized with 2% isoflurane and positioned on a table in the left lateral decubitus position, followed by measurement with a high-frequency ultrasound imaging system at 30 MHz (FUJIFILM VisualSonics, Canada). The left atrial diameter (LAD) was detected and recorded. Measurements were averaged over at least three cardiac cycles (22).

### Histological analysis

After measurement of cardiac function, the left atrial tissues were resected and fixed in 10% formalin, embedded with paraffin, and cut into 4 μm-thick samples. The cardiac morphology and fibrosis were detected using first hematoxylin and eosin (HE) staining, followed by Masson’s trichrome staining. After Masson’s trichrome staining, the collagen fibers are stained blue, and the cardiomyocytes are stained red. 

### Terminal deoxynucleotidyl transferase dUTP nick end labeling (TUNEL) assay

The apoptosis of cardiomyocytes was analyzed with a TUNEL assay using a commercial kit (Beyotime, China) following the manufacturer’s protocol. In brief, the tissue sections were treated with digoxigenin-dNTP and terminal deoxynucleotidyl transferase, after which the fluorescein-labeled anti-digoxigenin was administered. The nuclei were counterstained with DAPI. Five images of positive staining were taken under a light microscope (Olympus, Japan). 

### Atrial fibroblasts and cardiomyocytes isolation and culture

Atrial fibroblasts and cardiomyocytes were isolated from the hearts of newborn mice. Cardiac tissues were minced into small pieces and digested with 0.25% trypsin. The resulting cell suspensions were centrifuged and resuspended in DMEM that contains 10% fetal bovine serum (FBS), 100 µg/ml penicillin, and 100 μg/ml streptomycin in a 37 °C incubator filled with 5% CO_2_. Atrial fibroblasts were isolated by selectively removing cardiomyocytes through the adhesion of nonmyocytes (23). 

### Western blot analysis

Total protein extraction was obtained from cells and cardiac tissues using RIPA lysis buffer and estimated with a BCA assay kit (Beyotime, China). A total of 50 μg protein was divided using SDS-polyacrylamide gel electrophoresis (SDS-PAGE) and blotted to nitrocellulose (NC) membranes. The membranes were then blocked with 5% non-fat milk for two hours at room temperature and incubated with primary antibodies at 4 °C overnight, followed by secondary anti-rabbit or anti-mouse antibodies for one hour. The protein bands were visualized after incubation with an enhanced chemiluminescence reagent (Millipore, Germany).

### Quantitative real-time polymerase chain reaction (qPCR) analysis

Total RNA was isolated from fresh atrial tissues using TRIzol (Invitrogen, USA) according to the manufacturer’s protocol. The RNA was reverse-transcribed into single-stranded cDNA using the First-Strand Synthesis Kit (Takara, Japan). qPCR was performed using SYBR Green Premix (Takara, Japan) to examine gene expression levels. β-actin was employed as an internal control, and relative gene expression was determined using the 2^−ΔΔCT method. Primer sequences used for qPCR analysis were designed based on published sequences and confirmed using NCBI Primer-BLAST. The genes of interest examined included circFryl, miR-338-3p, TIMP4, and β-actin. 

Primer sequences used were TIMP4 plasmids (5’-UUUGUUAUUUUUGAAAUGCUGGA-3’), miR-338-3p (3’-GUUGUUUUAGUGACUACGACCU-5’), and small interfering RNA for circFryl (5’-UCUUCUCGACACUAUAUGCUGGG-3’). Sangon Biotech (Shanghai, China) synthesized and designed all primers, and the annealing temperature was optimized.

### Cell counting kit 8 (CCK-8) assay

Cell viability was detected using the CCK-8 assay kit (SolarBio, China). Cells (5 × 10^3^ cells/well) were seeded into a 96-well plate. The next day, exosomes (5 µg/ml) were added to each well and incubated for 24 hr. The absorbance was detected at 450 nm using a microplate reader (Perkin Elmer, Germany). 

### Cell apoptosis

After treatment, atrial fibroblasts and cardiomyocytes were collected, suspended in a detection buffer containing Annexin V and PI reagent (Beyotime, China), and reacted at 37 °C for 30 min. They were then washed with PBS, resuspended in 300 µl PBS, and detected with a flow cytometer (Beckman, USA). 

### Statistical analysis

The data are expressed as mean ± standard error of the mean (SEM). Statistical analysis was conducted using SPSS version 22.0 software. Differences between two or more groups were analyzed using Student’s t-test or one-way analysis of variance (ANOVA) followed by Bonferroni correction. The significance with *P*<0.05 was considered statistically significant.

## Results

### Identification of ADSC-exosomal circFry1

**Figure 1:**
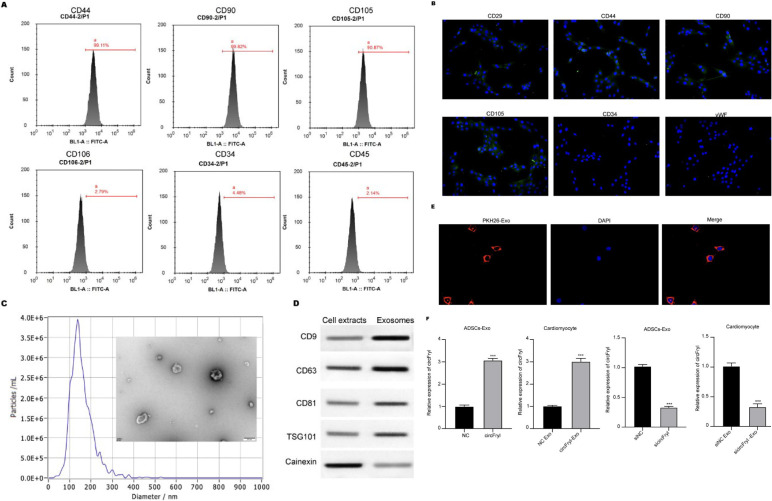
Identification of ADSC-exosomal circFry1

ADSCs were isolated from adipose tissues. The results from flow cytometry and immunofluorescence staining showed that isolated cells positively expressed CD44, CD90, CD105, and CD29 and negatively expressed CD106, CD34, CD45, and vWF, which is consistent with the features of stem cells (Figures 1A and 1B). Then, exosomes were isolated and identified from the culture medium of ADSCs. The TEM images indicated the sphere morphology of exosomes (Figure 1C), and the diameters mainly distribute around 150 nm (Figure 1C). The ADSC-exosomes presented high levels of CD9, CD63, CD81, and TSG101 and low calnexin level compared with the ADSCs, indicating the successful isolation of exosomes (Figure 1D). The PKH26-labeled exosomes could be successfully uptaken by cardiomyocytes (Figure 1E). After identification of the ADSC-exosome, we detected the delivery of circFryl by exosomes. As shown in Figure 1F, overexpression of circFryl in ADSCs notably elevated the level of circFryl in exosomes and the myocytes that were incubated with exosomes, whereas knockdown of circFryl exhibited the opposite effects. 

### ADSC-exosomal circFryl alleviates AF in vivo

**Figure 2:**
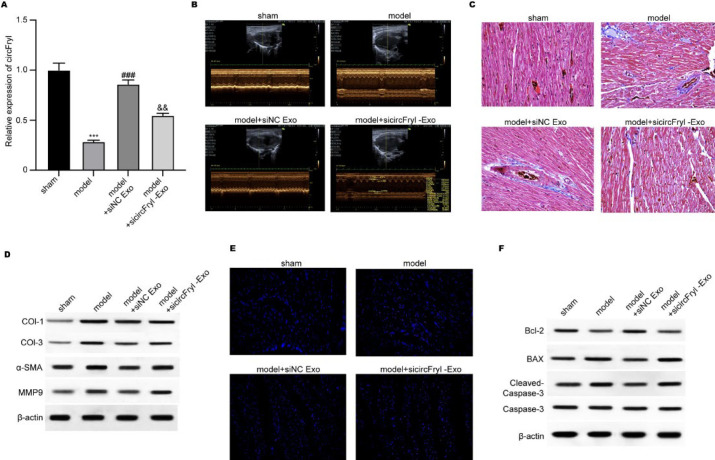
ADSC-exosomal circFryl alleviates AF *in vivo*

We established an *in vivo* model to mimic AF and administered exosome treatment; then, cardiac function was examined by echocardiographic measurement. As shown in Figures 2A and B, the cardiac tissues from the model group exhibited a relatively low level of circFryl and elevated left atrial diameter compared with the control group. Treatment with ADSC-exosomes significantly elevated circFryl level and recovered left atrial diameter, whereas knockdown of circFryl in exosomes abolished these effects. Moreover, cardiac fibrosis was measured by Masson’s trichrome staining and expression of fibrosis-related proteins. The levels of collagen fibers were much more significant in cardiac tissues of the AF model group than those of the control group (Figure 2C), supported by the up-regulation of fibrosis markers, such as collagen I, collagen III, α-SMA, and MMP9 (Figure 2D). ADSC-exosomes-treated cardiac fibrosis and deposition of collagen decreased significantly. However, the protective action was attenuated when circFryl-depleted ADSC-derived exosomes were applied. Results from the TUNEL experiment showed that ADSC-exosomes alleviated cardiac apoptosis of AF mice, whereas depletion of circFryl abolished the anti-apoptotic effects of ADSC-exosomes (Figure 2E). Consistently, ADSC-exosomes up-regulated the anti-apoptotic Bcl-2 expression and down-regulated the pro-apoptotic BAX and cleaved caspase-3 levels, which were abolished by circFryl depletion (Figure 2F). 

### ADSC-exosomal circFryl modulates the function of atrial fibroblasts and cardiomyocytes

**Figure 3:**
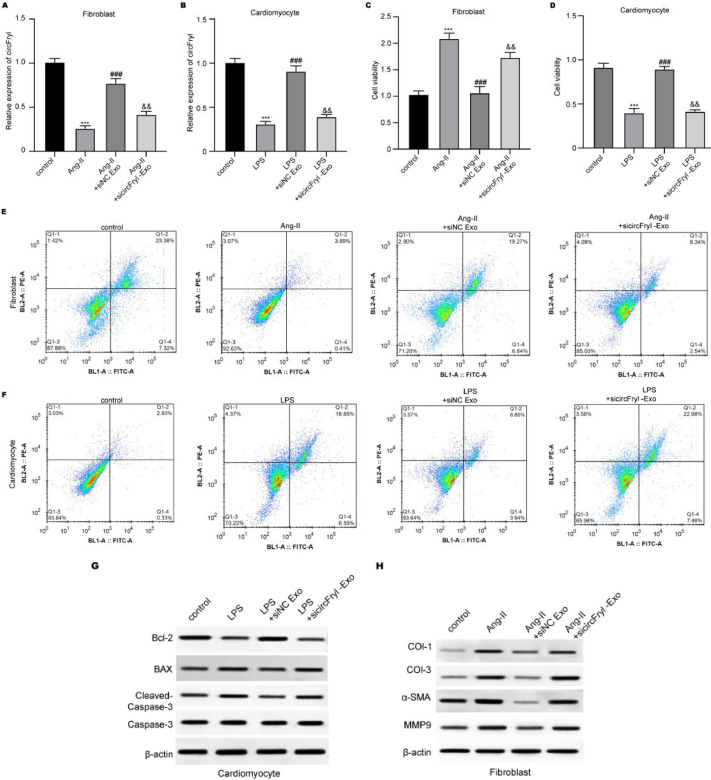
ADSC-exosomal circFryl modulates the function of atrial fibroblasts and cardiomyocytes

Subsequently, we measured the *in vitro* function of circFryl using angiotoninase II (Ang-II)-stimulated fibroblasts and lipopolysaccharides (LPS)-stimulated cardiomyocytes. ADSC-exosomes recovered the circFryl level in fibroblasts and cardiomyocytes under Ang-II and LPS stimulation, whereas circFryl-depleted exosomes successfully reduced circFryl level (Figure 3A and B). ADSC-exosomes treatment suppressed viability and enhanced cell apoptosis in Ang-II-induced fibroblasts, which was reversed by depletion of circFryl (Figures 3C and E). In contrast, ADSC-exosomal circFryl enhanced viability and suppressed apoptosis of LPS-induced cardiomyocytes (Figures 3D and F). Moreover, the expression of pro-apoptotic Bcl-2 was elevated, and pro-apoptotic BAX and cleaved caspase-3 levels were reduced by ADSC-exosomes, which was reversed by knockdown of circFryl (Figure 3G). The fibrosis-related collagen I, collagen III, α-SMA, and MMP9 levels were suppressed in Ang-II-stimulated fibroblasts, and knockdown of circFryl abolished the effects of ADSC-exosomes (Figure 3H). 

### ADSC-exosomal circFryl targets miR-338-3p to regulate TIMP4 expression

**Figure 4:**
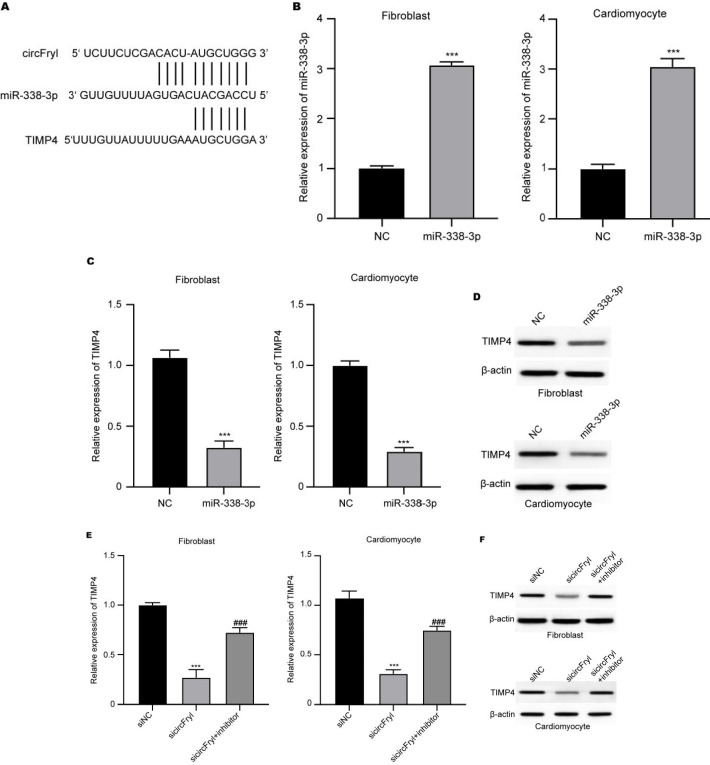
ADSC-exosomal circFryl targets miR-338-3p to regulate TIMP4 expression

Online analysis of miRNA interaction targets showed potential binding between miR-338-3p with circFryl and TIMP4 mRNA (Figure 4A). Next, we verified the circFryl-miR-338-3p-TIMP4 axis in fibroblasts and cardiomyocytes. Transfection with miR-338-3p significantly up-regulated the miR-338-3p level (Figure 4B) and down-regulated TIMP RNA and protein levels (Figures 4C and D). Besides, knockdown of circFryl notably suppressed TIMP level, and inhibition of miR-338-3p recovered TIMP level (Figures 4E and F). 

### ADSC-exosomal circFryl/miR-338-3p/TIMP4 axis modulates in vitro and in vivo AF function

**Figure 5:**
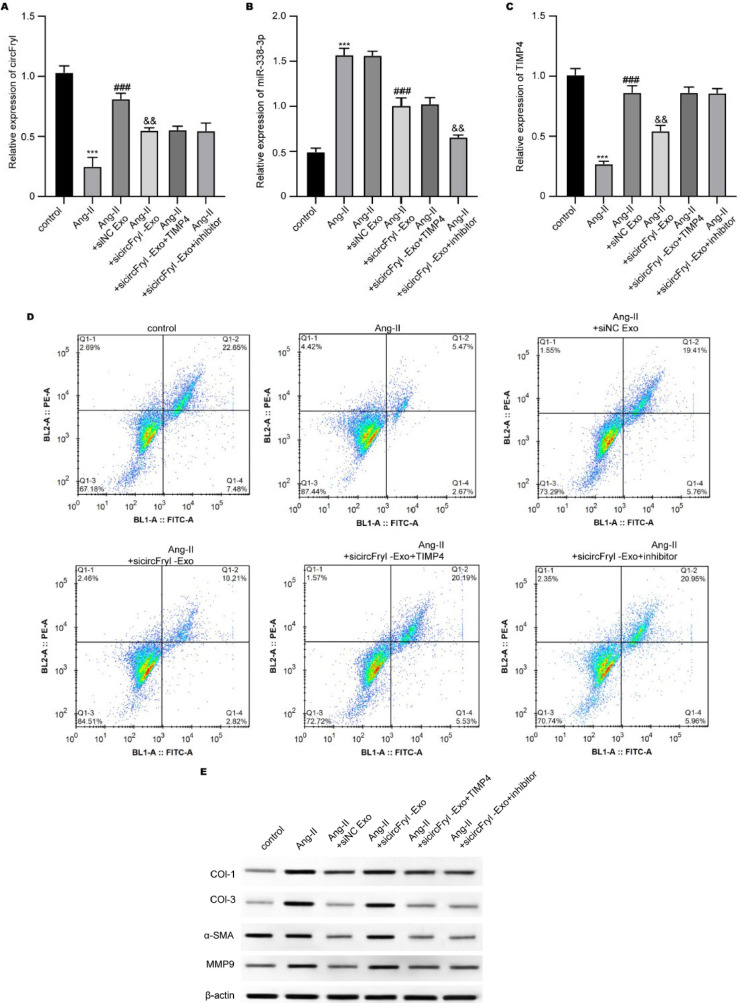
ADSC-exosomal circFryl/miR-338-3p/TIMP4 axis modulates fibroblast function

**Figure 6:**
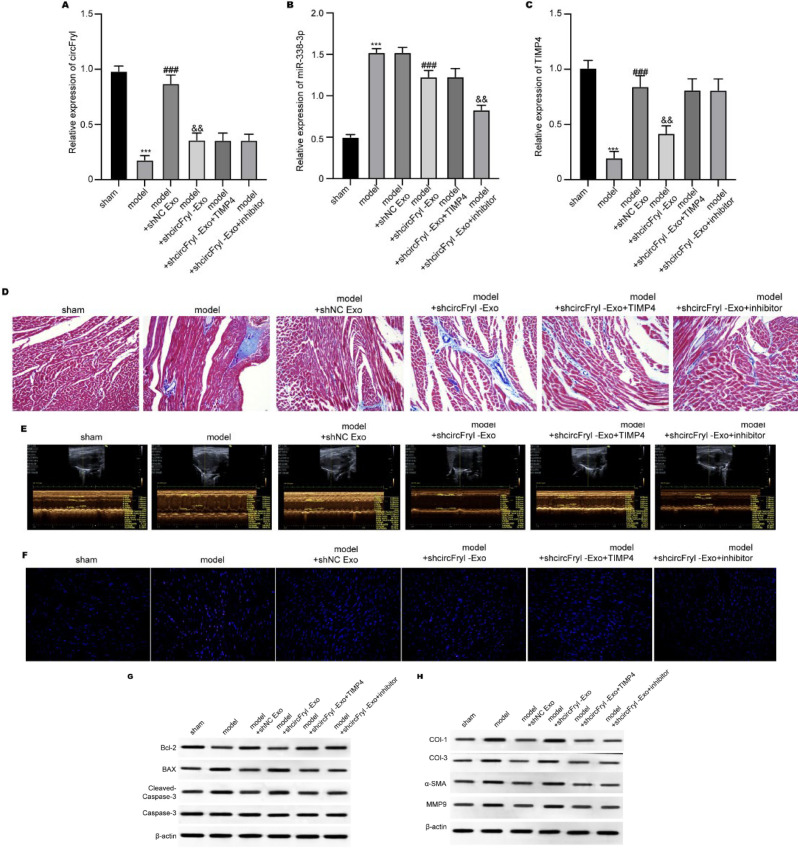
ADSC-exosomal circFryl modulates AF function via miR-338-3p/TIMP4 axis

To further verify the role of the circFryl-miR-338-3p-TIMP4 axis during AF, we administered TIMP4 overexpression and miR-338-3p inhibition *in vitro* and *in vivo*. The TIMP4 overexpression and miR-338-3p inhibition did not affect circFryl level (Figures 5A and 6A) but notably recovered TIMP4 expression in sicircFryl-treated fibroblasts (Figures 5B and C) and cardiac tissues from AF model (Figures 6B and C). The knockdown of circFryl reduced fibroblast apoptosis, whereas treatment with miR-338-3p inhibitor and TIMP4 overexpression elevated fibroblast apoptosis (Figure 5D) and suppressed the production of collagen I, collagen III, α-SMA, and MMP9 (Figure 5E). Furthermore, the ADSC-exosomes alleviated cardiac fibrosis (Figure 6D), repressed left atrial diameter (Figure 6E), and alleviated apoptosis (Figure 6F) in the AF model, which were reversed by circFryl depletion, whereas TIMP4 overexpression and miR-338-3p inhibition abolished the effects of sicircFryl (Figure 6D–F).

## Discussion

AF is a disturbed heart rhythm involving rapid and irregular atrial contractions. We demonstrated that ADSC-derived exosomes rescue AF mice from cardiac injury and dysfunction. Mechanistic studies showed that ADSC-derived exosomal circFryl suppresses miR-338-3p, resulting in the up-regulation of TIMP4 expression in cardiomyocytes and atrial fibroblasts. This, subsequently, enhances cardiomyocyte proliferation and suppresses cardiac fibrosis (24). 

In recent years, non-coding RNAs (ncRNAs) have emerged as key players in the pathogenesis of various diseases, including AF (24, 25). The ncRNAs play a critical role in the transcriptional and post-transcriptional regulation of genes, which could greatly benefit the interpretation of the AF pathogenesis (26). It has been reported that Atrial myocyte-derived exosomal microRNA contributes to atrial fibrosis in AF (27). Notably, circRNAs are representative forms of ncRNAs and usually function through the ceRNA (competitive endogenous RNA) mechanism, during which the ncRNAs competitively interact with specific miRNAs to interrupt the binding between miRNA and targeted mRNA (28, 29). Meanwhile, Exosomal circular RNAs play critical roles in cardiovascular diseases (30). Previous studies have indicated the regulatory effects of several circRNAs on AF pathogenesis. For example, Zhong and colleagues constructed and verified a ceRNA regulatory network associated with atrial fibrosis, which includes the up-regulated circ_0000672 and circ_0003916, down-regulated miR-516a-5p, and up-regulated hub genes such as KRAS and SMAD2 (31). The circ_0005019 exerted inhibitory effects on the proliferation and migration of cardiac fibroblasts and protected cardiomyocytes via functioning as the miR-499-5p sponge to modulate the expression of Kcnn3 (32). Moreover, a recent study reported that ADSC-derived exosomes showed high levels of the circFryl, and down-regulation of circFryl repressed the protective effects of ADSC-exosomes against sepsis-induced lung injury through suppressing inflammation and apoptosis (33). Consistent with these previous reports, our data confirmed that the knockdown of circFryl in ADSC-derived exosomes repressed the protective effects of ADSC-exosomes on AF-related cardiac fibrosis and cardiomyocyte death. 

Furthermore, we identified that the ADSC-exosomal circFryl acts as the ceRNA of miR-338-3p to up-regulate TIMP4 expression in fibroblasts and cardiomyocytes. The TIMP4 is a widely recognized inhibitor of matrix metalloproteinases (MMPs) and functions through direct interaction with MMPs to prevent their activation. It has been widely reported that TIMP4 is involved in the modulation of extracellular matrix degradation under normal conditions, and the overexpression of TIMP4 leads to the activation of signaling cascades that correlate with inflammation and fibrosis (34). Moreover, the involvement of TIMP4 in atrial fibrosis of patients with cardiac diseases has been extensively studied (35-39). Nevertheless, further experiments on the interaction between the circFryl with miR-338-3p and TIMP4 mRNA are needed. 

We applied bioinformatics tools in this work to predict and confirm crucial molecular interactions of ADSC-exosomal circFryl activity. Through publicly available databases such as TargetScan, miRDB, and StarBase, we identified miR-338-3p as a potential circFryl target, with TIMP4 serving as the downstream target. The predicted binding sites were also validated by existing reports on miRNA-mRNA interaction networks. Further, functional enrichment analysis of Gene Ontology (GO) and Kyoto Encyclopedia of Genes and Genomes (KEGG) databases points out that miR-338-3p is associated with pathways regulating extracellular matrix remodeling and cardiac fibrosis, which are vital in AF pathophysiology. These bioinformatics-derived findings and our experimental verifications enhance the mechanistic involvement of the circFryl/miR-338-3p/TIMP4 axis in ADSC-exosome-protected cardioprotection. Combining computational predictions with *in vitro* and *in vivo* experiments gives a complete picture of this regulatory network and its possible therapeutic applications.

(A) Identification of ADSC surface biomarkers by flow cytometry. (B) Identification of ADSC surface biomarkers by immunofluorescence staining. (C) Morphology and particle size of exosomes. (D) Markers of exosomes detected by Western blotting. (E) Exosomes were labeled with PKH26 and internalized by cardiomyocytes. (F) The level of circFryl in ADSCs and ADSC-derived exosomes. ***P<0.001. ADSCs: Adipose-derived stem cell

(A) Level of circFryl in cardiac tissues was measured using qPCR. (B) Echocardiographic measurement of left atrial diameter. (C) Masson’s trichrome staining of cardiac tissues. (D) Expression of collagen I, collagen III, α-SMA, and MMP9 in cardiac tissues was detected by western blotting assay. (E) TUNEL staining of apoptotic cells. (F) Expression of apoptosis-related proteins in cardiac tissues. ADSCs: Adipose-derived stem cell; AF: Atrial fibrillation

The fibroblasts were induced by Ang-II, and cardiomyocytes were stimulated by LPS. (A and B) The circFryl level in fibroblasts and cardiomyocytes was measured by qPCR assay. (C and D) The viability of fibroblasts and cardiomyocytes was detected by CCK-8 assay. (E and F) The cell apoptosis was determined using flow cytometry. (G) Expression of apoptosis-related proteins in cardiomyocytes was detected using western blotting assay. ***P<0.001 vs control, ###*P*<0.001 vs LPS or Ang-II, &&*P*<0.01 siNC exo. ADSCs: Adipose-derived stem cell

(A) The predicted interaction between miR-338-3p with circFryl and TIMP4 mRNA. (B-D) The fibroblasts and cardiomyocytes were transfected with miR-338-3p mimics, then (B) miR-338-3p level, (C) TIMP4 RNA, and (D) TIMP4 protein levels were measured. (E and F) The fibroblasts and cardiomyocytes were transfected with sicircFryl and miR-338-3p inhibitors. The RNA and protein levels of TIMP4 were detected. ***P<0.001 vs control, ###*P*<0.001 vs LPS. ADSCs: Adipose-derived stem cell

The fibroblasts were induced by Ang-II and treated with ADSC-exosomes, TIMP4 overexpression, and miR-338-3p inhibitors. (A-C) The levels of circFryl, miR-338-3p, and TIMP4 were measured by qPCR assay. (D) Fibroblast apoptosis was determined by flow cytometry. (E) Expression of collagen I, collagen III, α-SMA, and MMP9 was detected by Western blotting assay. ***P<0.001 vs control, ###*P*<0.001 vs Ang-II, &&*P*<0.01 siNC exo. ADSCs: Adipose-derived stem cell

(A-C) The levels of circFryl, miR-338-3p, and TIMP4 in cardiac tissues were measured by qPCR assay. (D) Masson’s trichrome staining of cardiac tissues. (E) Echocardiographic measurement of left atrial diameter. (F) TUNEL staining of apoptotic cells. ***P<0.001 vs control, ###*P*<0.001 vs Ang-II, &&*P*<0.01 siNC exo. ADSCs: Adipose-derived stem cell; AF: Atrial fibrillation

## Conclusion

ADSC-derived exosomes could deliver circFryl to cardiac fibroblasts and cardiomyocytes and subsequently interact with the miR-338-3p/TIMP4 axis to modulate TIMP4 expression. Our findings present the circFryl/miR-338-3p/TIMP4 axis as a novel molecular mechanism and target for the protective effects of ADSC-exosomes on AF. 

## Data Availability

Any additional datasets generated during and/or analyzed during our current investigation that can support this study’s findings are available from the corresponding author upon reasonable request.

## Declaration

We have not used any AI tools or technologies to prepare this manuscript.

## Animals Studies

All institutional and national guidelines for the care and use of laboratory animals were followed and approved by the Ethics Committee of Xinghua People’s Hospital.
